# Engagement Mediates the Relationship Between Emotion and Achievement of Chinese EFL Learners

**DOI:** 10.3389/fpsyg.2022.895594

**Published:** 2022-07-05

**Authors:** Enhao Feng, Gang Hong

**Affiliations:** ^1^College of Foreign Languages, Zhejiang Normal University, Jinhua, China; ^2^School of English Studies, Zhejiang International Studies University, Hangzhou, China

**Keywords:** positive psychology (PP1.0 and PP2.0), achievement emotions, engagement, foreign language enjoyment, foreign language classroom anxiety

## Abstract

Since the coming of Positive Psychology in the field of second language acquisition (SLA), the significance of emotion, especially positive emotion, has been well recognized by researchers. Educational research has indicated that both emotion and engagement play fundamental roles in learning process and psychological wellbeing, but research on their relationship is scant in SLA. The present study contributed to the development of Positive Psychology in SLA by investigating the relationships between achievement emotions, behavioral engagement, and self-reported English achievements. 633 students from four senior high schools in China participated in the study. Data collected through questionnaires revealed: (1) Participants reported a medium to high level of foreign language enjoyment (FLE), foreign language classroom anxiety (FLCA), and behavioral engagement; females had a higher level of FLE and behavioral engagement than males; the participants reported more anxiety and less enjoyment than western samples; (2) significant correlations were found between FLE, FLCA, behavioral engagement, and self-reported achievement; (3) significant mediating effects of behavioral engagement were found between both achievement emotions and self-reported achievement, and the mediating effect of engagement was stronger between FLCA and self-reported achievement. The findings extended the nomological network of achievement emotions, developed our insights into the complex relationships between emotions, learner engagement and language achievement, and revealed the mediating effects of behavioral engagement. Finally, directions for future study and implications for foreign language educators were provided.

## Introduction

Neuroscience and cognitive science have revealed the fundamental role of emotion in learning process (Pekrun and Linnenbrink-Garcia, 2012). Although the field of second language acquisition (SLA) has long been dominated by research on learners’ cognitive factors (Sharwood Smith, 2017), changes happened rapidly in the past decade (Dewaele et al., 2019). Emotion is now attracting increasing research attention. A review of the literature revealed the complicated relationships between emotions and learner-related variables, such as gender, grade and language achievement (Dewaele and MacIntyre, 2014; Dewaele et al., 2018; Jiang and Dewaele, 2019; Li, 2020). Earlier in SLA, anxiety was almost the only emotion being studied (Horwitz et al., 1986), however, in the past decade, under the influence of positive psychology (PP), a “positive turn” is emerging in the field, and more positive emotions (e.g., enjoyment) are studied (Oxford, 2015).

Although relevant research is accumulating, the nomological network of emotion is yet to be fully explored (Botes et al., 2021). For instance, the relationship between emotion and engagement is unclear. Though both emotion and engagement are the crucial components of psychological well-being (Seligman, 2018), engagement has received insufficient attention in SLA. The significance of engagement was highlighted as the “bridge” connecting teaching and learning, without which meaningful learning is unlikely to occur (Hiver et al., 2021). Engagement, as a result of personal factors such as emotions, is also critical in understanding achievements (Oga-Baldwin, 2019). Previous educational research has investigated the simple bivariate relationship between emotion and engagement (Do and Schallert, 2004). However, according to Oga-Baldwin’s (2019) contextual model, engagement could mediate the influence of emotions on achievement, functioning as the mechanism underlying the relationship between emotion and language learning achievement. Nevertheless, relevant empirical studies are scant in SLA. It is thus urgent to investigate the relationship between engagement, achievement emotions, and achievement in foreign language (FL) learning, and also the underlying mechanism of their relationships.

Given the research gaps, the present study aimed to investigate the relationships between emotions, behavioral engagement, and FL achievement, and also the mediating effect held by behavioral engagement in the relationships. Specifically, the goal of the current study was to reveal: (1) the general levels of achievement emotions (i.e., enjoyment and anxiety) and behavioral engagement of Chinese senior high school FL learners, and the relative level of enjoyment and anxiety in the present sample compared with the western sample; the gender differences in enjoyment, anxiety, and behavioral engagement; (2) the correlations between enjoyment, anxiety, behavioral engagement and FL self-reported achievement (SRA); (3) the mediating effects of behavioral engagement between achievement emotions (i.e., enjoyment and anxiety) and FL self-reported achievements. The findings may narrow the research gap in the field by extending the nomological network of achievement emotions in SLA, and providing original insights into the complex relationships between achievement emotion, engagement, and learning outcomes, especially the mediating effects of behavioral engagement between achievement emotion and FL achievement. A better understanding of the relationships could provide implications for a more accurate intervention to improve learners’ engagement, wellbeing and FL learning achievement.

## Literature Review

### Foreign Language Classroom Anxiety and Foreign Language Enjoyment

Anxiety is the most investigated achievement emotion in SLA (MacIntyre, 2017). Horwitz et al. (1986) obtained feedbacks from FL learners about anxiety-inducing stimuli in their FL classes. All the reported sources of FL anxiety together formed a “conceptually distinct variable in FL learning” (p. 125), namely FLCA, which is defined as “a distinct complex of self-perceptions, beliefs, feelings, and behaviors related to classroom learning arising from the uniqueness of the language learning process” (Horwitz et al., 1986 p. 128). Since then, research on FLCA was burgeoning, which generally revealed that under most circumstances this emotion has negative effects on language learning, such as worsened cognition and achievement (Gardner et al., 1997; Horwitz, 2001; MacIntyre, 2002), and negative attitudes toward the language (Dewaele, 2005). Surprisingly, although FLCA has been investigated for decades, empirical studies rarely investigated its relationship with engagement, which is a big gap to be narrowed.

Influenced by PP, especially the so-called second wave of positive psychology (i.e., PP 2.0; MacIntyre, 2021), more studies taking a holistic view, focusing on both positive (e.g., enjoyment) and negative emotions (e.g., anxiety) were conducted (e.g., Dewaele and MacIntyre, 2014; Jiang and Dewaele, 2019; Li et al., 2019; Dewaele and Li, 2021). Foreign Language Enjoyment (FLE) is probably the most investigated positive emotion in SLA. Compared with FLCA which was already frequently studied in previous research (Horwitz, 2001, etc.), studies on FLE just began several years ago (Dewaele and MacIntyre, 2014). Most studies on FLE were directed by the broaden-and-build theory (Fredrickson, 1998) and control-value theory (Pekrun, 2006). One of the pioneering research on FLE is Dewaele and MacIntyre (2014), which found that there was a moderate correlation between FLE and FLCA with only 13% variance shared, suggesting a degree of inter-relationship but essentially independence of the dimensions. Their moderate and negative correlation was confirmed in numerous studies in different contexts (Dewaele and Alfawzan, 2018; Dewaele et al., 2018; Jiang and Dewaele, 2019; Li, 2020). Moreover, the authors (Dewaele and MacIntyre, 2014) found that FLE was positively correlated with students’ self-perceived English as a foreign language (EFL) proficiency, and the relationship was negative between FLCA and self-perceived EFL proficiency. This finding was replicated later in many studies (e.g., Dewaele et al., 2018; Li et al., 2019). Dewaele and MacIntyre (2014) found that learners felt more FLE than FLCA, a pattern that was also confirmed in many later studies (Dewaele et al., 2018; Dewaele and MacIntyre, 2019; Jiang and Dewaele, 2019). However, as noted by Dewaele (2021), this pattern may not be generalizable to the whole learner population because the data were collected through snowball or convenience sampling. Therefore, more research should be conducted in various contexts to check the generalizability of this pattern.

Gender differences in FLE and FLCA were found in previous research, with females typically feeling higher levels of both FLE and FLCA than males (e.g., Dewaele and MacIntyre, 2014; Dewaele et al., 2016). Nevertheless, some studies conducted in the Chinese context reported inconsistent findings, for instance, the gender difference of FLE and FLCA was insignificant in Jiang and Dewaele (2019). In Li (2022) the gender difference of FLE was insignificant. Therefore, more research is needed to have a closer look at the gender effect on FLE and FLCA in the Chinese EFL learning context.

The effect size of the influence of FLE and FLCA on FL achievement can differ. For instance, Dewaele and Alfawzan (2018) found that the effects of FLE and FLCA on foreign language performance were different, indicating that the positive effect of FLE was stronger than the negative effect FLCA has on FL performance.

SLA studies on positive emotions are also emerging in China. Li et al. (2018) is perhaps the earliest systematic study on FLE in China, in which the authors adopted the 14-item version of the FLE scale (Dewaele and MacIntyre, 2016) in the Chinese EFL context to examine its psychometric properties. They further produced an 11-item Chinese Version of the Foreign Language Enjoyment Scale and identified a 3-factor structure, namely FLE-private, FLE-teacher, and FLE-environment. Qualitative data collected through open questions showed that the individual experience of FLE is also shaped by a large range of learner-internal and learner-external variables besides the effect of teacher and peers. Later, the same authors (Li et al., 2019) investigated the relationship between FLE, FLCA and EFL achievements, and found that FLE and FLCA was moderately and negatively correlated, and FLE was positively related to achievements, while FLCA negatively related to achievements. Their results largely replicated findings in Dewaele and MacIntyre (2014).

Recently, Li (2020) investigated the mediating effect of FLE in the relationship between trait emotional intelligence and self-perceived and actual EFL achievement. The author found that FLE significantly and positively influenced both self-perceived and actual EFL achievement, and FLE partially mediated the influence of trait emotional intelligence on both self-perceived and actual achievement.

Despite that emotion studies are flourishing in SLA, the research scope is still limited, and more learner-internal or -external factors should be taken into consideration, for example, engagement. Although previous studies suggested that the indicators of engagement could be numerous, including emotion (Oga-Baldwin, 2019), relevant research is still scarce in SLA, which hence necessitates the present study.

### Engagement

Engagement is said to be a “new kid on the block” compared with popular construct such as motivation (Reschly and Christenson, 2012, p. 14). Maybe this explains why only a limited number of studies have investigated engagement in SLA. The significance of engagement, however, had already been recognized in the field of education, in which a wave of interest in engagement is emerging (Hiver et al., 2021), and related research is accumulating (Fredricks et al., 2019). Engagement has even been described as ‘the holy grail of learning’ (Sinatra et al., 2015, p. 1), and was found to be a significant predictor of learners’ academic achievement.

There are two main frameworks for investigating engagement in SLA, namely Svalberg’s (2009, 2017, 2021) framework of engagement with language (EWL), and Philp and Duchesne’s (2016) model of task engagement. Svalberg (2007, 2009, 2017) defined language engagement as “a cognitive, and/or affective, and/or social state and a process in which the learner is the agent and the language is the object and maybe the vehicle (means of communication)” (Svalberg, 2009, p. 244). Three dimensions are identified in EWL: cognitive, social, and affective, all of which were found to be interdependent and correlated (Svalberg, 2018). Engagement is defined by Philp and Duchesne (2016, p. 51) as “a state of heightened attention and involvement, in which participation is reflected not only in the cognitive dimension, but in social, behavioral, and affective dimensions as well.” In Philp and Duchesne’s (2016) model of task engagement, four facets are featured, namely behavioral, cognitive, social, and affective.

As Skinner et al. (2009, p. 778) suggested, “the core construct, most prototypical of engagement, is behavioral participation in the classroom,” while the cognitive and agentic engagement can be considered as a subcomponent of behavioral engagement (Skinner et al., 2009). The same was also argued by Oga-Baldwin (2019, p. 5): “… engagement in class at least partially begins with behavior, and the other parts of the process, including cognition, agency, and emotion, all result in part from students’ initial, subconscious decision to engage or disengage behaviorally.” Both of the abovementioned frameworks are superior to previous conceptualizations which are mostly single-dimensioned because the two frameworks are both more comprehensive and fine-grained (Dao et al., 2021). However, the task-based framework (Philp and Duchesne, 2016) includes a behavioral dimension of engagement, which is a core dimension but is absent in EWL. Hence the present study adopted the task-based framework of engagement (Philp and Duchesne, 2016), and focused on behavioral engagement.

Behavioral engagement is reflected by the amount and quality of learners’ active participation in learning, and early second language (L2) research operationalized behavioral engagement by gauging word counts and turn counts (Dörnyei and Kormos, 2000; Platt and Brooks, 2002; Bygate and Samuda, 2009). In L2 learning, for example, learners’ behavioral engagement includes learners’ voluntary involvement in speaking, interactional initiative, time on task, the amount of semantic content produced while on task, and persistence on task without the need for support or direction (Philp and Duchesne, 2016). However, since almost all engagement includes some degree of action, more recent studies operationalize behavioral engagement as ‘students’ expenditure of effort on learning tasks, the quality of their participation, and their degree of active involvement in the learning process’ (Sang and Hiver, 2021). Engagement has another important feature, namely domain-specificity (Wang et al., 2016), indicating that it varies according to contexts and subjects (Sinatra et al., 2015). That is to say, engagement must be investigated separately and the scale measuring it must also be specialized, which again highlights the necessity and significance of investigating engagement in SLA.

Educational research has indicated that engagement is a strong predictor of academic achievement (Fredricks et al., 2004). For instance, Collie et al. (2017) employed a structural equation modeling (SEM) based on a sample of 186 undergraduate students, and found that negative behavioral engagement was negatively correlated with general academic achievement. Wang and Holcombe (2010) collected data from 1,046 urban students and conducted a SEM, finding that behavioral engagement positively influence general academic achievement. Recently, Quintero et al. (2022) investigated the relationship between math anxiety, three dimensions pf engagement (i.e., cognitive-behavioral, emotional, and social), and math achievement, and found that behavioral-social engagement positively influenced math achievement, but the other two kinds of engagement negatively influenced math achievement. However, how would behavioral engagement function in SLA and influence EFL achievement is relatively less investigated, therefore, more research is needed.

### Foreign Language Enjoyment, Foreign Language Classroom Anxiety, and Engagement

Engagement is a result of both environmental facilitators such as classroom interpersonal relations, and personal factors such as motivation and emotions. Indeed, Oga-Baldwin (2019) proposed that affective factors could influence learner engagement in SLA. Although FLE and FLCA have been studied in a variety of contexts, there is little research in SLA, to the author’s knowledge, that has investigated the relationships between FLE, FLCA, and behavioral engagement, let alone the underlying mechanism of their relationships.

Recently, Dewaele and Li (2021) conducted a mixed-method study to investigate the interconnections among perceived teacher enthusiasm, student emotions (enjoyment and boredom), and social-behavioral engagement. In their study, 2,002 EFL learners from 11 universities in China were involved. The results revealed a complex interaction between the three factors. Mediation analysis revealed that teacher enthusiasm as perceived by learners, directly and indirectly, affected social-behavioral learning engagement. The indirect link went through enjoyment and boredom. Both emotions were linked to teacher enthusiasm, positively for enjoyment, and negatively for boredom, and these emotions mediated the effect of students’ perceived teacher enthusiasm on their own engagement. However, their focus was social-behavioral engagement rather than behavioral engagement as the present study investigated.

Probably the only quantitative study that directly investigated the relationship between FLE, FLCA, and engagement is Khajavy (2021), which examined the correlation between FLE, FLCA, grit, engagement, and L2 reading achievement in the L2 learning context. The results suggested that FLE positively predicted engagement. FLCA was not a significant predictor of engagement. Moreover, FLCA was found to be significantly and negatively correlated with L2 reading achievement, and perseverance was a positive predictor of L2 reading achievement. The mediating role of engagement was also found between perseverance and L2 reading achievement, interest and L2 reading achievement, and L2 enjoyment and L2 reading achievement.

In Guz and Tetiurka (2016), the authors investigated the teacher-related factors that contribute to positive emotions and learner engagement in FL young learners (6−12 years old). Their results revealed that a positively oriented, cognitively, and emotionally engaged teacher elicits similar mindset in learners, which could, in turn, spark engagement. Some pedagogical practices were also found to facilitate learners’ engagement. Learners were found to be engaged: when the activity they are performing is set in a familiar context/when the activity offers an opportunity for personalization/when the activity provides them with a feeling of mastery and competence/when there is evidence of carefully planned scaffolding (Guz and Tetiurka, 2016). However, the sample size in this study was too small, and its qualitative nature could also limit the findings from being generalized to larger populations.

### Engagement as a Mediator

Previous research has indicated that engagement is a potential mechanism by which achievement emotions influence academic achievement. According to the broaden-and-build theory and previous empirical studies (e.g., Fredrickson, 1998, 2013; Dewaele and MacIntyre, 2014; Dewaele and Li, 2021), positive emotions can broaden people’s mind scope and build cognitive as well as social resources, which in turn boost engagement. At the same time, many educational studies have found the positive influence of engagement on learning achievement (Park, 2005; Ponitz et al., 2009; Wang and Holcombe, 2010; Collie et al., 2017). The prerequisites for mediation are that an independent variable predicts a mediator variable and that the mediator variable simultaneously predicts a dependent variable (Pekrun et al., 2009). Indeed, Oga-Baldwin’s (2019) contextual model of engagement suggested that achievement emotion could influence the next time’s achievement through the functioning of learner engagement. Several educational studies also found the mediational role of behavioral engagement between achievement emotions and academic achievement. One case in point is Quintero et al. (2022), which conducted a SEM and found the mediating effect of three kinds of engagement (i.e., cognitive-behavioral, emotional, and social) between math anxiety and math achievement. Another example is Kwon et al. (2018), which found the mediating effect of behavioral engagement in the relationship between negative emotionality and reading achievement, and between emotion regulation and reading achievement. Combined, they indicated that emotions may influence achievement indirectly through the mediation of behavioral engagement.

The present study hence sat out to narrow the research gaps by examining the relationships between FLCA, FLE, behavioral engagement, and EFL self-reported achievement (SRA) in the context of Chinese high school EFL learning. The present study also aimed to examine behavioral engagement as a mediator between achievement emotions (i.e., FLE and FLCA) and SRA.

## Methodology

### Research Questions

The specific research questions that directed the present study were as follows:

RQ1: What are the general levels of FLE, FLCA, and behavioral engagement of the participants? Do participants’ levels of FLE and FLCA differ from their western peers? Are there any gender differences in FLE, FLCA, and behavioral engagement?

RQ2: What are the relationships between their FLE, FLCA, behavioral engagement, and EFL self-reported achievements?

RQ3: Does behavioral engagement mediate the relationships between achievement emotions and EFL self-reported achievements?

### Participants

The participants of the current study were 633 EFL learners from four Chinese senior high schools in a city in Zhejiang Province, China, with a convenience sampling being adopted. Two of the schools were second-tier high schools, one first-tier high school and the other one a third-tier high school. The four schools were chosen because they represent participants with varied academic levels so that the findings may be more representative and generalizable. All of the participants were Chinese native speakers with their ages ranging from 15 to 20, and the mean was 16.4 (*SD* = 1.05). There were 279 males (44.08%) and 354 females (55.92%). Most of them were year one (*n* = 302, 47.7%) and year three students (*n* = 304, 48.3%), and only 27 were year two students (4.3%). English was one of the major subjects included in the National College Entrance Examination (NCEE). They had one English class per day, an English morning self-study class every 2 days, each English session took 45 mins.

### Instruments

The instrument used in this research was a 5-point Likert composite questionnaire. The questionnaire started with a demographics section requiring information including gender, age, and grade. Following this were three sub-scales, in which participants were asked to rate their attitude toward the items from “1” (very unfavorable) to “5” (very favorable). The three sub-scales consisted of the Foreign Language Enjoyment (FLE) scale (Li et al., 2018), Foreign Language Classroom Anxiety (FLCA) scale (Dewaele and MacIntyre, 2014), and behavioral engagement scale (Zhou et al., 2021). At the bottom of the questionnaire was a part measuring learner’s self-reported English achievements.

Enjoyment was measured through the FLE scale. It was a modified version of the FLE scale in Dewaele and MacIntyre (2016) adapted by Li et al. (2018) based on Chinese samples. It had been demonstrated to have high internal reliability (Cronbach’s α = 0.83, *n* = 11), and validity. This scale contained 11 items (e.g., “Our English teacher is friendly.”), in which 3 dimensions had been identified, namely FLE-teacher, FLE-private, and FLE-environment. In the present study, the scale also had high reliability (Cronbach’s α = 0.89; McDonald’s ω = 0.89; *n* = 11). According to Hu and Bentler (1999), an acceptable CFA model should have an RMSEA (Root Mean Square Error of Approximation) lower than 0.08, a CFI (Comparative Fit Index), a TLI (Tucker-Lewis Index) larger than 0.9, and an SRMR (Standardized Root Mean Square Residual) lower than 0.08. Therefore, the model fit of FLE scale in the present study was good [χ^2^ (40) = 196.074; RMSEA = 0.079; CFI = 0.96; TLI = 0.945; SRMR = 0.054].

The FLCA scale was a shortened scale extracted by Dewaele and MacIntyre (2014) from Horwitz et al.’s (1986) original scale. It measured learners’ anxiety specific to the FL classroom. The scale was more concise and remained highly reliable (Cronbach’s α = 0.89; McDonald’s ω = 0.89; *n* = 8) as the original one. The scale was recently validated by Botes et al. (2022). There were 8 items in the scale with a single dimension (e.g., “Even if I am well prepared for English class, I feel anxious about it.”), and two of the items were reversed (e.g., “I feel confident when I speak in English class.”). A scale analysis revealed high internal reliability of this scale in the current study (Cronbach’s α = 0.84, McDonald’s ω = 0.84; *n* = 8). The scale had good CFA model fit [χ^2^ (17) = 39.2; RMSEA = 0.045; CFI = 0.986; TLI = 0.978; SRMR = 0.032].

Behavioral engagement was measured through the behavioral engagement scale (Zhou et al., 2021). There was only one factor in the scale with 8 items (e.g., “I put effort into learning in my language class.”). Three of the items were reverse worded (e.g., “I don’t participate much in my language class.”). Minor adjustments were made to make it suitable for the EFL learning context (specifically, “In my English class…” was added to the beginning of each statement). Its reliability was acceptable in the present study (Cronbach’s α = 0.82; McDonald’s ω = 0.82; *n* = 8). Its model fit was good after deleting an item with factor loading lower than 0.40 (“I do other things in the language classroom when I am supposed to be paying attention.”) [χ^2^ (13) = 32.902; RMSEA = 0.054; CFI = 0.979; TLI = 0.967; SRMR = 0.027].

The final part was a 5-point Likert scale asking participants to report their conceptualizations about their English standings in the classroom. Items include: 1 = bad, 2 = below average, 3 = average, 4 = above average, 5 = good. Self-reported achievement rather than actual scores was elicited for several reasons: first, previous research has confirmed a high concurrent validity between self-reported academic achievement scores and actual scores (Kuncel et al., 2005; Cole and Gonyea, 2010). Self-reported scores turned out to rank as the highest factor that correlated with the actual academic performance (Hattie, 2009). Second, the participants were from different schools, which designed and administered different English exams, resulting in different tests across the schools, hence actual test scores may not be comparable across the four schools. Third, since the data collection happened at the beginning of the term when participants didn’t have any tests yet, using self-reported rather than a detailed actual score could lessen learners’ cognitive burden of recalling their test scores in the last semester so as to increase the response rate.

The questionnaire had good composite reliability (CR) with McDonald’s ω = 0.81. The questionnaire was finished anonymously in a traditional paper-and-pencil way due to the ban on mobile phone use in the school.

Since there were already Chinese versions of the FLE and FLCA scale (Li et al., 2018; Jiang and Dewaele, 2019), they were adopted with minor adjustments. The behavioral engagement scale (Zhou et al., 2021) was translated into Chinese by the first author, and then a bilingual postgraduate student majoring in applied linguistics back-translated it into English to check the appropriacy. Finally, the first author discussed with a professor in applied linguistics to formulate the final version.

### Procedures

Before the study, the author got permission from the headmasters of all schools, and consent was obtained from all the participants as well as their parents. The questionnaires were distributed by the headteacher of each class. Participants were briefly informed of the purpose of the study and were told that their personal information will be strictly protected. 15 mins were given to fill out the questionnaires. As soon as they finished, the headteacher collected the questionnaires then handed them over to the author. Finally, 647 students volunteered in the study, and 14 invalid questionnaires were discarded because of incomplete responses, producing a final sample of 633 participants, so the response rate was 97.8%.

### Data Analysis

SPSS 26.0, jamovi 2.0, and Mplus 8.3 (Muthén and Muthén, 2018) were used to analyze the data. First of all, the distribution of data was checked through a combination of both visual methods (e.g., Q-Q plot) and normality tests (e.g., Shapiro-Wilk test) as recommended by many (Peat and Barton, 2008; Field, 2009; Ghasemi and Zahediasl, 2012). The results indicated that the data well fitted the normal distribution (see Supplementary Material). Hence the use of parametric statistics was justified. Before any further analysis, a series of scale analyses were employed through jamovi 2.0 to check the internal reliability of scales. Then three separate Confirmatory Factor Analysis (CFA) were conducted through Mplus to check whether the constructs fitted the data. After the validation of instruments, the descriptive statistics were generated, and an independent sample *t*-test and a one sample *t*-test were conducted for RQ 1. A series of Pearson correlation analyses were adopted for RQ 2. The multiple regression analysis and PROCESS v 3.4.1 (Model 4) (Hayes, 2013) mediation analysis were employed for RQ3.

## Results

### Preliminary Analysis

As Table 1 indicated, participants’ FLE, FLCA, and behavioral engagement were at a medium to a high level. To compare FLE with FLCA levels, average scores on the 5-point scale were calculated, based on which a paired samples *t*-test was conducted. Surprisingly, the result revealed that there is no significant difference between FLE and FLCA (M_*FLE*_ = 3.36, M_*FLCA*_ = 3.35; *t* = 0.165, *p* = 0.869).

**TABLE 1:** Descriptive of the general levels of achievement emotions and behavioral engagement.

Variable	Theoretical range	Middle point	Mean	*SD*	Min	Max	Skewness (SE)	Kurtosis (SE)
FLE	11−55	33	37	7.09	15	54	−0.30 (0.10)	0.10 (0.20)
FLCA	8−40	24	26.8	6.26	8	40	−0.22 (0.10)	−0.17 (0.20)
Engagement	7−35	21	25.8	4.21	14	35	−0.05 (0.10)	−0.23 (0.19)

Independent samples *t*-tests were performed to check if there were gender differences in the participants. Results revealed a significant gender difference in FLE and behavioral engagement. Female learners felt significantly more enjoyment than their male peers (*t* = 5.303, *p* < 0.001, Cohen’ s d = 0.42), and their behavioral engagement was also significantly higher than that of their male classmates (*t* = 4.73, *p* < 0.001, Cohen’ s d = 0.38). However, in the present study no statistically significant gender difference was found in FLCA (*t* = 0.57, *p* = 0.57, Cohen’s d = 0.05).

A one-sample *t*-test was conducted to check if there is any statistically significant difference between the present sample and the western sample in Dewaele et al. (2018), which was conducted on 189 western FL learners from two English high schools with their ages ranging from 12 to 18. Their study (Dewaele et al., 2018) used the adapted version of the FLE scale in Dewaele and MacIntyre (2016) which was largely the same as the one used in the present study, and their FLCA scale was totally the same with the present one, hence ensuring the comparability. The results revealed a significant difference in FLE, with the FLE level of the present sample significantly lower than that in Dewaele et al. (2018) (M_1_ = 3.36, *SD* = 0.65; M_2_ = 3.9, *SD* = 0.6; *t* = −21.1, *p* < 0.001, Cohen’s d = −0.84), and the FLCA level in the present study was significantly higher than that of the western sample (M_1_ = 3.35, *SD* = 0.78; M_2_ = 2.4, *SD* = 0.8; *t* = 30.6, *p* < 0.001, Cohen’s d = 1.22).

### The Bivariate Correlations Between Foreign Language Enjoyment, Foreign Language Classroom Anxiety, Behavioral Engagement, and Self-Reported Achievements

A series of Pearson correlation analyses were conducted to reveal the bivariate correlations among the observed variables. The results were summarized in Table 2.

**TABLE 2:** The bivariate correlations between achievement emotions, behavioral engagement, and self-reported achievement.

	FLE		FLCA		Engagement		SRA
FLE	—						
FLCA	−0.33	***	—				
Engagement	0.59	***	−0.35	***	—		
SRA	0.43	***	−0.38	***	0.33	***	—

***p < 0.001, FLE, foreign language enjoyment; FLCA, foreign language classroom anxiety; SRA, self-reported achievement.

As shown in Table 2, there were significant correlations between all the key variables. FLE was negatively and significantly correlated with FLCA (*r* = −0.33, *p* < 0.001), suggesting that learners with higher enjoyment tended to feel less anxiety in the EFL classroom. FLE was positively and significantly correlated with behavioral engagement (*r* = 0.59, *p* < 0.001), indicating that learners with higher enjoyment were also more behaviorally engaged in learning activities. FLCA was found to be negatively related to behavioral engagement (*r* = −0.35, *p* < 0.001), suggesting that more anxious learners tended to be less engaged in learning activities. FLE was significantly and positively correlated with SRA (*r* = 0.43, *p* < 0.001), while FLCA was negatively correlated with their SRA (*r* = −0.38, *p* < 0.001). This suggested that learners with more enjoyment were likely to perceive themselves as having better achievement in English, while students with more anxiety tended to perceive themselves as less proficient in English. Besides, behavioral engagement was also reported to be significantly and positively correlated with SRA (*r* = 0.33, *p* < 0.001), indicating that those more engaged learners tended to be more confident in their learning achievements and perceive themselves as more proficient in English.

### Mediating Effects of Behavioral Engagement Between Achievement Emotions and Self-Reported Achievements

To answer the final research question, the mediating effect of behavioral engagement between achievement emotions (i.e., FLE and FLCA) and SRA was investigated. In the model 1, FLE was the antecedent variable X, and SRA was proposed to be the consequent variable Y, while behavioral engagement was the mediating variable M. In the model 2, FLCA was the antecedent variable X, while the mediating variable and consequent variable were the same as the first model. SPSS PROCESS v 3.4.1 (Model 4; Hayes, 2013) was used to estimate the mediating effects. Since previous studies (Dewaele and MacIntyre, 2014) suggested that gender and grade could have a significant influence on FLE and FLCA, they were controlled in both the regression analyses and mediation analyses.

To test the first model, linear regression analysis and PROCESS with 5,000 bootstrap samples were conducted, offering corresponding 95% confidence intervals. The results were as follows (Tables 3, 4).

**TABLE 3:** Regression results of model 1.

Regression equations		Fit index			Coefficient	
Outcome	Predictor	*R*	*R* ^2^	*F*	β	*T*
SRA		0.47	0.22	60.56***		
	FLE Grade Gender				0.39 0.12 0.15	10.83*** 3.38*** 4.22***
Engagement		0.59	0.35	112.39***		
	FLE Grade Gender				0.57 0.02 0.07	17.31*** 0.50 2.02*
SRA		0.48	0.23	47.23***		
	FLE				0.33	7.58***
	Engagement Grade Gender				0.10 0.12 0.14	2.42** 3.34*** 4.03***

β are standardized coefficients, **p < 0.01, ***p < 0.001, FLE, foreign language enjoyment; SRA, self-reported achievement.

**TABLE 4:** Analysis of mediating effect in model 1.

	Effect size	Boot SE	Boot LLCI	Boot ULCI	Percentage of effect
Mediating effect	0.09	0.04	0.01	0.17	15.25%
Direct effect	0.50	0.07	0.37	0.63	84.75%
Total effect	0.59	0.05	0.48	0.70	100%

*Boot SE, the bootstrap standardized error; Boot LLCI, lower level of confidence interval; Boot ULCI, upper level of confidence interval.*

As Table 3 indicated, FLE predicted all the outcome variables from a modest effect size (0.1 < β < 0.3), a moderate effect size(0.3 < β < 0.5), to a strong effect size (β > 0.5) (Cohen et al., 2011). When gender and grade were controlled, FLE significantly and positively influenced SRA (β = 0.39, *p* < 0.001); FLE also influenced learner’s behavioral engagement significantly and positively (β = 0.57, *p* < 0.001); when put into the same regression model, engagement influenced learner’s SRA significantly and positively (β = 0.10, *p* < 0.01), and FLE’s positive influence on SRA was still significant (β = 0.33, *p* < 0.001) though the effect diminished after engagement being entered into the model.

**FIGURE 1:**
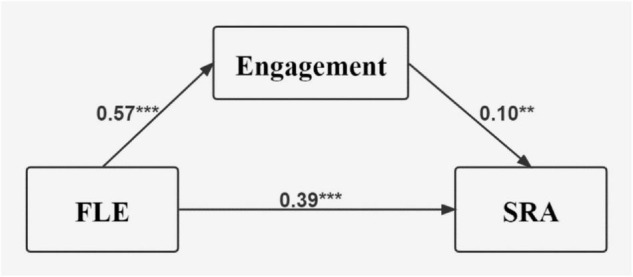
The mediation model 1. All are standardized coefficients, **p < 0.01, ***p < 0.001, FLE, foreign language enjoyment; SRA, self-reported achievement.

As Table 4 showed, the 95% confidence interval of the indirect effect of behavioral engagement did not straddle zero, suggesting that there was indeed a mediating effect of behavioral engagement between FLE and SRA, and the mediating effect took up 15.25% of the total effect of FLE on SRA. The variance inflation factor (VIF = 1.56) was slightly above 1, indicating that the possibility of multicollinearity was marginal. The first mediation model was hence as Figure 1 demonstrated.

To test the second model, the same steps as did in model 1 were followed. The regression analyses results were shown in Table 5.

**TABLE 5:** Regression results of model 2.

Regression equations		Fit index			Coefficient	
Outcome	Predictor	R	R^2^	F	β	t
SRA		0.47	0.22	57.86***		
	FLCA Grade Gender				−0.37 0.12 0.24	−10.48*** 3.28* 6.81***
Engagement		0.40	0.16	39.53***		
	FLCA Grade Gender				−0.35 0.03 0.19	−9.46*** 0.88 5.24***
SRA		0.49	0.24	50.61***		
	FLCA				−0.31	−8.29***
	Engagement Grade Gender				0.18 0.11 0.21	4.78*** 3.16* 5.81***

β are standardized coefficients, *p < 0.05, ***p < 0.001, FLCA, foreign language classroom anxiety; SRA, self-reported achievement.

As Table 5 demonstrated, FLCA predicted all the outcome variables with modest to moderate effect sizes. FLCA significantly and negatively influenced SRA (β = −0.37, *p* < 0.001), and it also negatively and significantly influenced behavioral engagement (β = −0.35, *p* < 0.001). When put into the same model, FLCA still influenced SRA significantly and negatively (β = −0.31, *p* < 0.001), though the effect diminished, and behavioral engagement positively and significantly influenced SRA (β = 0.18, *p* < 0.001). The positive effect of FLE on both behavioral engagement (β = 0.57) and SRA (β = 0.39) was found to be stronger than the negative effect of FLCA on behavioral engagement (β = −0.35) and SRA (β = −0.37).

**FIGURE 2:**
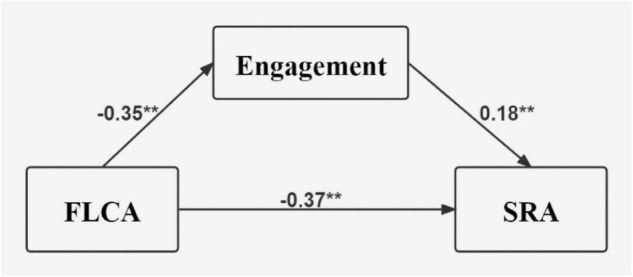
The mediation model 2. All are standardized coefficients, **p < 0.01, FLCA, foreign language classroom anxiety; SRA, self-reported achievement.

The results of mediation analysis was shown in Table 6, the 95% confidence interval of the indirect effect of behavioral engagement didn’t straddle zero, indicating that the indirect effect of behavioral engagement between FLCA and SRA was significant, with 17.40% of the total effect taken by behavioral engagement as the mediating effect. The mediation model 2 was visualized as Figure 2. The mediating effect taken up by behavioral engagement was larger between FLCA and SRA than that between FLE and SRA. The model was visualized as Figure 2. The variance inflation factor (VIF = 1.189) was slightly above 1, indicating that the possibility of multicollinearity was small. According to the effect size, engagement played a bigger mediational role between FLCA and SRA than between FLE and SRA.

**TABLE 6:** Analysis of mediating effect in model 2.

	Effect Size	Boot SE	Boot LLCI	Boot ULCI	Percentage of Effect
Mediating Effect	−0.08	0.02	−0.12	−0.04	17.40%
Direct Effect	−0.38	0.05	−0.48	−0.30	82.60%
Total Effect	−0.46	0.04	−0.55	−0.38	100%

*Boot SE, the bootstrap standardized error; Boot LLCI, lower level of confidence interval; Boot ULCI, upper level of confidence interval.*

## Discussion

The first research question concerns the general levels of FLE, FLCA, and behavioral engagement; the gender and international differences in FLE, FLCA, and behavioral engagement, and international differences in FLE and FLCA. Our participants reported a medium to a high level in FLE, FLCA, and behavioral engagement. Interestingly, we found participants’ levels of FLE and FLCA did not differ significantly. This finding is quite different from most previous studies (e.g., Dewaele and MacIntyre, 2014; Jiang and Dewaele, 2019), and may indicate that the pattern found in Dewaele and MacIntyre (2014), namely FLE is generally higher than FLCA, is not universal. A significant gender difference was found in FLE and behavioral engagement in the present study. The female participants had significantly higher levels of FLE and behavioral engagement than their male peers, but the difference was not significant in FLCA. Female participants’ higher level in FLE echoes some previous studies (Dewaele and MacIntyre, 2014, 2016; Dewaele et al., 2018). The result partially confirms previous findings that women tend to show more internalizing emotions and positive emotions (Chaplin and Aldao, 2013). But in the present study, female participants seemed to only show a heightened level of FLE with the absence of a significantly higher level of FLCA than their male peers, which contradicts some studies (e.g., Jiang and Dewaele, 2019). The abovementioned gender difference may be explained by the fact that female students care more about their FL performance and engage more in FL learning perhaps because they find learning the FL language intrinsically appealing for them (Dörnyei and Csizér, 1998; Henry and Cliffordson, 2013). When compared with western FL learners, our participants had significantly higher FLCA level and lower FLE level, with both effect sizes bigger than 0.7, indicating middle to large effect sizes (Plonsky and Oswald, 2014, p. 889). This result echoes previous findings (Jiang and Dewaele, 2019; Li et al., 2019; MacIntyre et al., 2019), indicating that Chinese EFL learners generally felt more anxiety and less enjoyment than western FL learners. Reasons for this can be multifaceted. The first reason could be the test-oriented atmosphere in Chinese high schools. Since English is one of the three major subjects in Gaokao (National College Entrance Examination, namely NCEE), it was highly valued by students themselves, their teachers, and families. A high-stake test like NCEE could have a significant impact on student’s future career, social status and salary, hence learning English could be more than just learning a foreign language. Students are under both learning and social pressure, which could make them feel more anxious than their western peers. Additionally, the linguistic distance could be an alternative explanation of the difference. Previous research (Botes et al., 2021) found that FL learners whose native language was more linguistically similar to the target language feel less anxiety and more enjoyment. The FL learners in Dewaele et al. (2018) mostly had Indo-European languages as their mother tongue (e.g., Spanish, French, Germany), hence the linguistic distance was relatively smaller than that between English and Chinese. Because English belongs to the Indo-European language family while Chinese belongs to Sino-Tibetan languages. However, it should be noted that although the study (Dewaele et al., 2018) we used to compare adopted a very similar FLE scale with the present one, they were not totally the same. Therefore, the interpretation could be weakened, at least to some degree, by the difference of measurement.

The second research question is about the correlations between FLE, FLCA, behavioral engagement, and SRA. The results indicated a negative correlation between FLE and FLCA, a positive relationship between FLE and behavioral engagement, and a negative relationship between FLCA and behavioral engagement. SRA was found to be positively correlated with FLE and behavioral engagement, and negatively with FLCA. The findings are in accordance with previous studies (e.g., Wang and Holcombe, 2010; Reschly and Christenson, 2012; Dewaele and MacIntyre, 2014; Collie et al., 2017; Li et al., 2019; Wang et al., 2019).

The third research question concerns the mediating effects of behavioral engagement between FLE, FLCA, and SRA. We first found that FLE positively influenced behavioral engagement and SRA, and FLCA negatively affected behavioral engagement and SRA. Our findings indicated that the positive broadening effects of FLE on both behavioral engagement and SRA were stronger than the negative narrowing effects of FLCA on behavioral engagement and SRA, thus dovetailing with previous research (Dewaele and Alfawzan, 2018). The present study further revealed that behavioral engagement significantly mediated the influence of both FLE and FLCA on EFL self-reported achievement, echoing previous studies (e.g., Kwon et al., 2018; Oga-Baldwin, 2019; Quintero et al., 2022). In other words, learners who felt more enjoyment tended to be more behaviorally engaged in learning activities, which in turn positively influenced their SRA. On the contrary, anxious learners tended to behaviorally disengage from learning activities and have a lower SRA. This could be interpreted by the broaden-and-build theory (Fredrickson, 1998, 2001, 2013). According to the broaden-and-build theory, positive emotions can broaden thought-cognition-action, while negative ones could narrow it (Fredrickson, 1998). Enjoyment could broaden learners’ resources cognitively and behaviorally, which could, in turn, give learners more resources to get engaged in learning tasks. A higher engagement indicated a more active participation in learning activities and interactions such as asking and answering questions, which would, in turn, promote higher achievement (Wang and Holcombe, 2010). While FLCA, as a negative emotion, could narrow learners’ resources, which would, in turn, limit learners’ cognitive and behavioral resources, hence it could be harder for them to get engaged in learning, leading to poorer SRA.

Most previous SLA research only investigated the simple and bivariate relationships between achievement emotions and other factors (e.g., Jiang and Dewaele, 2019). The present study hence contributed to the literature by revealing the complex mediating effect of engagement in the influence of achievement emotions on language achievement. The present findings highlighted the significance of engagement in foreign language learning. This study also empirically confirmed that engagement is indeed a product of personal factors such as emotions (Oga-Baldwin, 2019). The mediating effects of behavioral engagement may also imply that the relationship between emotions and learning outcomes is not that simple and direct, but is complex with many possible mediators and moderators.

## Pedagogical Implications, Limitations, and Future Directions

The findings of the present study had important implications for Chinese senior high school EFL educators. The present study found that emotions could directly and indirectly affect self-reported learning achievements through learners’ behavioral engagement. As MacIntyre and Mercer (2014, p. 156) suggested, language educators must first be aware of the significance of improving learners’ language learning experience. Then teachers should take measures to boost students’ positive emotions and mitigate negative ones, for instance, they can match task demands to learners’ capacities, because over-challenging tasks could elicit negative emotions and decrease positive ones (Pekrun, 2006). A previous study (Jang et al., 2010) indicated that teachers’ autonomy support positively predicted students’ engagement, hence teachers should give learners more autonomous support, and avoid “controlling” them. Moreover, many emotional methods proposed by PP have been found effective in containing anxiety, for instance, the ABCDE macro strategy (Seligman, 2006, 2011), which draws on the theory and practice of rational emotive behavior therapy (REBT; Ellis, 2003). Given that females seemed to have more enjoyment and engagement than males in the present study, teachers should also pay attention to male students’ emotions and engagement to prevent them from disengagement and low wellbeing in the FL classroom. Given the complex relationship between emotions, engagement and achievement, multiple methods could be adopted by teachers at every possible point. For example, at the very beginning of a class, teachers could ensure a positive learning environment as a good start of a class. During the class, teachers could organize activities that could try to maintain the learner’s positive emotions as well as engagement (e.g., leaving learners space to choose learning activities or use activities that are reachable for learners). However, total elimination of anxiety is both unnecessary and unrealistic, a good balance between enjoyment and anxiety is recommended (Fredrickson, 2013).

Despite the important implications the present study provided, there were some limitations in the present study that should be acknowledged. First, to ensure the comparability of FL achievements among different schools, the present study used self-reported achievement rather than actual achievement. Although self-reported achievement has been found to be highly related to actual achievement (Kuncel et al., 2005; Cole and Gonyea, 2010), it cannot represent actual achievement. Therefore, future research could use actual language achievements. Second, the research instrument in the present study was a composite questionnaire, while a questionnaire is unable to capture dynamic changes in achievement emotions and engagement, and it cannot reveal the fine-grained individual differences within participants. Future studies are recommended to adopt a mixed-method design and complement quantitative method with qualitative one. Finally, since the present study was a cross-sectional one, no causal relationships should be assumed in the present study. Future studies are encouraged to use experimental and longitudinal designs.

## Conclusion

The present study was one of the first attempts to investigate the complex relationships between FLE, FLCA, behavioral engagement, and EFL self-reported achievements of Chinese senior high school EFL learners. The results revealed that: (1) participants reported medium to a high level of FLE, FLCA, and behavioral engagement, and no significant difference was found between FLE and FLCA; female participants had higher levels of FLE and behavioral engagement than their male peers, however, the difference was not significant in FLCA; when compared with western students, our participants felt significantly more anxiety and less enjoyment; (2) behavioral engagement was found to be positively correlated with FLE and SRA, while was negatively correlated with FLCA; SRA was negatively correlated with FLCA, and positively correlated with FLE; FLE and FLCA was negatively correlated (3) behavioral engagement partially mediated the influence of FLE and FLCA on SRA, with the mediating effects between FLE and SRA weaker than that between FLCA and SRA.

The present study extended the nomological networks of achievement emotions and revealed the mediating role of behavioral engagement between achievement emotions and EFL self-reported achievement, which was not found in previous SLA studies. Our study highlighted the critical role of engagement in understanding learning achievements (Oga-Baldwin, 2019). The mediating effects may indicate that the relationship between emotion and learning outcomes is not simple as previous studies assumed.

## Data Availability Statement

The original contributions presented in this study are included in the article/supplementary material, further inquiries can be directed to the corresponding author/s.

## Ethics Statement

Ethical review and approval was not required for the study on human participants in accordance with the local legislation and institutional requirements. Written informed consent to participate in this study was provided by the participants’ legal guardian/next of kin.

## Author Contributions

EF contributed to the conceptualization, data analysis, and drafting of the manuscript. GH contributed to the conceptualization, critical revision, and proofreading of the article. Both authors contributed to manuscript revision, read, and approved the submitted version.

## Conflict of Interest

The author declares that the research was conducted in the absence of any commercial and financial relationships that can be construed as potential conflicts of interest.

## Publisher’s Note

All claims expressed in this article are solely those of the authors and do not necessarily represent those of their affiliated organizations, or those of the publisher, the editors and the reviewers. Any product that may be evaluated in this article, or claim that may be made by its manufacturer, is not guaranteed or endorsed by the publisher.
