# Supplementary figures and images for: Engagement Mediates the Relationship Between Emotion and Achievement of Chinese EFL Learners
Source: Front Psychol. 2022 Jul 5;13:895594. doi: 10.3389/fpsyg.2022.895594 (PMC9295811; doi:10.3389/fpsyg.2022.895594)

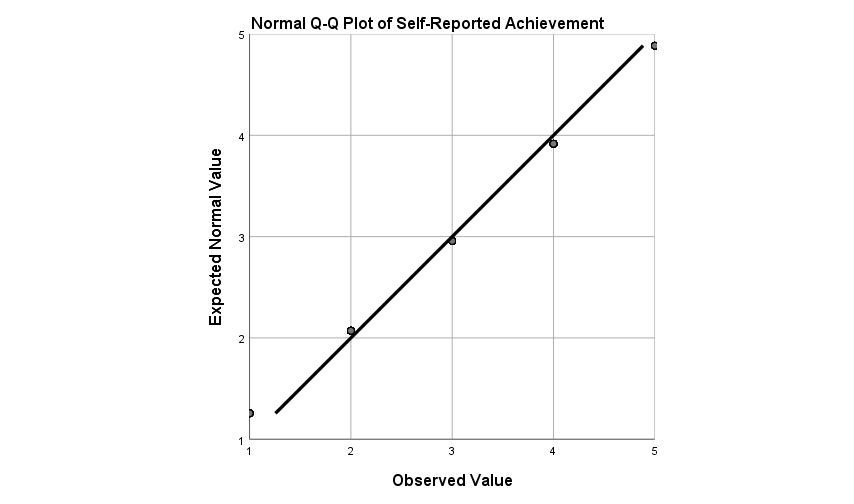

Supplement: Supplementary file 1 [file Image_1.png]

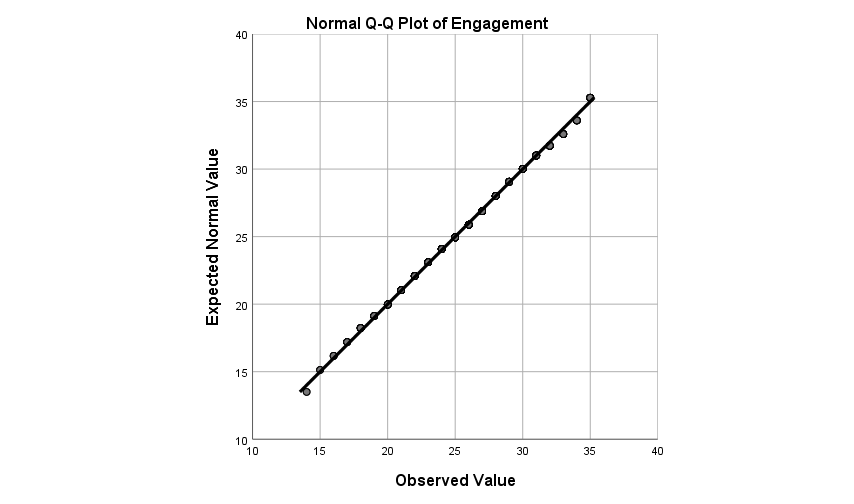

Supplement: Supplementary file 2 [file Image_2.png]

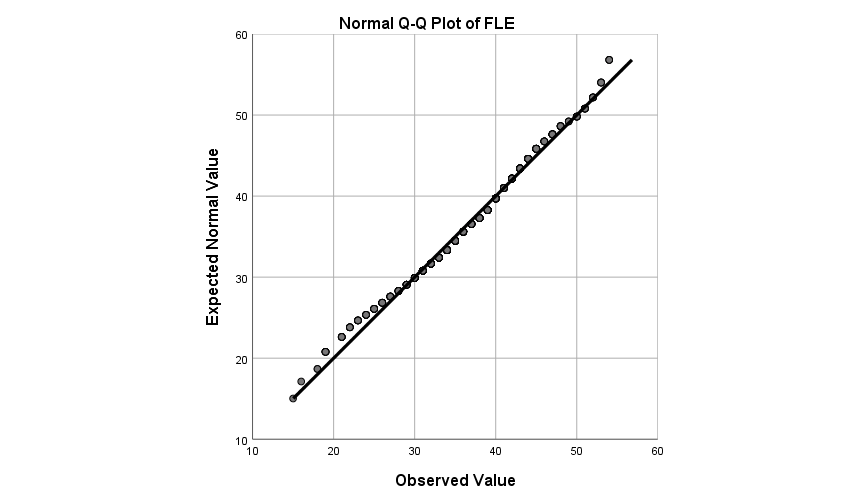

Supplement: Supplementary file 3 [file Image_3.png]

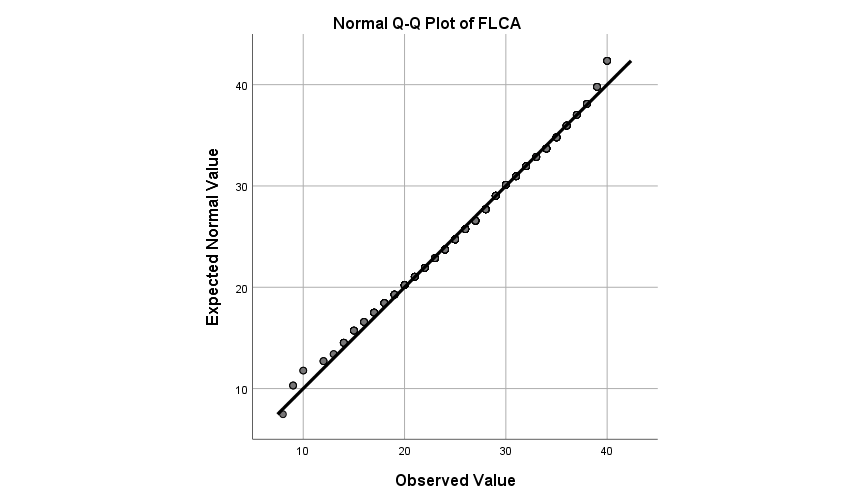

Supplement: Supplementary file 4 [file Image_4.png]
